# Effect of 3D Synthetic Microscaffold Nichoid on the Morphology of Cultured Hippocampal Neurons and Astrocytes

**DOI:** 10.3390/cells11132008

**Published:** 2022-06-23

**Authors:** Clara Alice Musi, Luca Colnaghi, Arianna Giani, Erica Cecilia Priori, Giacomo Marchini, Matteo Tironi, Claudio Conci, Giulio Cerullo, Roberto Osellame, Manuela Teresa Raimondi, Andrea Remuzzi, Tiziana Borsello

**Affiliations:** 1 Department of Pharmacological and Biomolecular Sciences, Università degli Studi di Milano, Via Balzaretti, 9, 20133 Milano, Italy; claraalice.musi@marionegri.it (C.A.M.); erica.priori@unimi.it (E.C.P.); 2 Mario Negri Insitute for Pharmacolgical Research—IRCCS, Via Mario Negri, 2, 20156 Milano, Italy; arianna.giani@marionegri.it (A.G.); giacomo.marchini@marionegri.it (G.M.); matteo.tironi@marionegri.it (M.T.); 3 Division of Neuroscience, IRCCS San Raffaele Scientific Institute, 20132 Milan, Italy; colnaghi.luca@hsr.it; 4 School of Medicine, Vita-Salute San Raffaele University, 20132 Milan, Italy; 5 Department of Chemistry, Materials and Chemical Engineering “G. Natta”, Politecnico di Milano, 20133 Milano, Italy; claudio.conci@polimi.it (C.C.); manuela.raimondi@polimi.it (M.T.R.); 6 Department of Physics, Istituto di Fotonica e Nanotecnologie (IFN)-CNR, Politecnico di Milano, 20133 Milano, Italy; giulio.cerullo@polimi.it (G.C.); roberto.osellame@polimi.it (R.O.); 7 Department of Management, Information and Production Engineering, University of Bergamo, Via Marconi, 5, 24044 Dalmine, Italy; andrea.remuzzi@unibg.it

**Keywords:** primary culture, dendritic spines, brain on dish, 3D co-cultures, brain models

## Abstract

The human brain is the most complex organ in biology. This complexity is due to the number and the intricate connections of brain cells and has so far limited the development of in vitro models for basic and applied brain research. We decided to create a new, reliable, and cost-effective in vitro system based on the Nichoid, a 3D microscaffold microfabricated by two-photon laser polymerization technology. We investigated whether these 3D microscaffold devices can create an environment allowing the manipulation, monitoring, and functional assessment of a mixed population of brain cells in vitro. With this aim, we set up a new model of hippocampal neurons and astrocytes co-cultured in the Nichoid microscaffold to generate brain micro-tissues of 30 μm thickness. After 21 days in culture, we morphologically characterized the 3D spatial organization of the hippocampal astrocytes and neurons within the microscaffold, and we compared our observations to those made using the classical 2D co-culture system. We found that the co-cultured cells colonized the entire volume of the 3D devices. Using confocal microscopy, we observed that within this period the different cell types had become well-differentiated. This was further elaborated with the use of drebrin, PSD-95, and synaptophysin antibodies that labeled the majority of neurons, both in the 2D as well as in the 3D co-cultures. Using scanning electron microscopy, we found that neurons in the 3D co-culture displayed a significantly larger amount of dendritic protrusions compared to neurons in the 2D co-culture. This latter observation indicates that neurons growing in a 3D environment may be more prone to form connections than those co-cultured in a 2D condition. Our results show that the Nichoid can be used as a 3D device to investigate the structure and morphology of neurons and astrocytes in vitro. In the future, this model can be used as a tool to study brain cell interactions in the discovery of important mechanisms governing neuronal plasticity and to determine the factors that form the basis of different human brain diseases. This system may potentially be further used for drug screening in the context of various brain diseases.

## 1. Introduction

The human brain is the most complex structure in biology [1,2]. This complexity is due to the high number of neuronal cells that constitute it and their intricate connections: the brain is formed by more than 100 billion neurons that form at least 100 trillion synapses [3]. This has so far limited the development of in vitro models for basic and applied brain research [4], forcing researchers to mainly rely on in vivo animal models with the associated technical and ethical issues [5]. This lack of models has also negatively impacted the development of treatments for the majority of neurological diseases [6,7,8].

Nevertheless, several strategies have been used to mimic some aspects of brain structure and function in in vitro conditions. In these models, particular attention was given to synapses, the junctions between neurons whose function forms the basis of processes such as learning and memory. Given the critical role synapses play in brain function, it is not surprising that synapse dysfunction and loss have a devastating effect on neuronal communication, underlying many psychiatric and neurologic diseases, such as mental retardation [9], schizophrenia [10], Parkinson’s disease [11], autism [12], Alzheimer’s disease [13], compulsive behavior [14], and drug addiction [15].

Ideally, to closely mimic the synaptic function in physiological and pathological conditions, in vitro models should include several aspects: (i) a comparable spatial distribution of brain cells in the tissue; (ii) the ability of neurons to form synaptic networks; and (iii) the presence of other groups of brain cells, including astrocytes. Astrocytes are particularly important at the synaptic level for their trophic support, the fine-tuning of the transmission, and their contribution to structural plasticity [16,17,18].

Conventional in vitro models, however, usually rely on two-dimensional (2D) cell culture substrate systems and have a limited capability to provide functional responses and spatial self-organization [4]. This is mainly due to the fact that cells modify their distribution dynamically on these substrates, generating a random network architecture [19,20]. This is caused by insufficient cell–cell and cell–extracellular matrix interactions [5,21,22]. In addition, neuronal networks are organized in three dimensions [23], a feature that is clearly lacking in the flat neuronal cultures within a dish [24,25,26].

Recent developments in laser technology have provided new materials that could potentially suit the requirements for in vitro models that mimic the soft brain tissue such as porosity, morphology, and surface topography [27]. The aim of this study is to adapt a previously described 3D synthetic microscaffold developed for cancer and stem cells to the culture of primary brain cells. A system that, among the several advantages, allows the 3D culture of cells with similar effort, costs, and reproducibility as the classic 2D cultures.

To this end, we optimized the 3D synthetic microscaffold Nichoid [28] for the co-culture growth of primary neurons and astrocytes. This scaffold technology has never been used for the culture of brain cells. In previous articles, the Nichoid has allowed the successful culture of mesenchymal cells and neuronal stem cells. Both of these cell types maintain mitotic activity in vitro and are therefore fundamentally different from the post-mitotic hippocampal mouse primary neurons. This difference requires the use of distinct experimental protocols to set stable and reproducible conditions that guarantee the right time plating of the brain cells and the correct ratio between astrocytes and neurons. Using various markers and morphological criteria, we demonstrated that similar to the in vivo conditions, neurons cultured in the Nichoid display mature spines and an increased amount of filopodia compared to 2D cultures. This important observation opens the possibility to take advantage of the Nichoid as an innovative tool for 3D brain modeling.

## 2. Materials and Methods

### 2.1. Laser Microfabrication

The synthetic microscaffolds called Nichoids were fabricated by means of two-photon polymerization (2PP) with the largely validated organic/inorganic negative biocompatible photoresist called SZ2080 [29,30]. The photoresist was mixed with a commercial photoinitiator, Irgacure-369 (Sigma-Aldrich, St. Louis, MO, USA), to obtain a more efficient polymerization process. To date, this material has shown no cytotoxicity and a low autofluorescence emission, providing the most suitable conditions for the cell culture and fluorescence microscope observations [31]. The fabrication setup employed a femtosecond laser system (femtoREGEN, High Q Laser GmbH, Rankweil, Austria) and a spatial light modulator (PLUTO NIR-049, HOLOEYE, Berlin, Germany) to generate a precise and predetermined intensity pattern in the focus of the objective, which allowed a fast multi-foci fabrication approach [31]. The laser beam was focused onto the sample with a 100× oil immersion microscope objective (Plan-Apochromat, Zeiss, Munich, Germany), and the sample was moved by three (X-Y-Z) brushless computer-controlled linear translators (ANT130, Aerotech, Pittsburgh, PA, USA). Prior to 2PP, the SZ2080 photoresist was drop-cast on circular glass coverslips (diameter = 12 mm, thickness ~ 175 μm, BioOptica, Milan, Italy), allowing solvent evaporation at room temperature for 48 h. Once irradiated, all the unpolymerized regions were removed by developing the samples in a 50:50 (*v*/*v*) methyl isobutyl ketone—isopropyl alcohol solution for 20 min. The cell culture substrate was fabricated by drilling the bottom of a 35 mm plastic Petri dish (EuroClone, Pero, Italy) in its central portion. The obtained hole had a diameter equal to the circular portion of the glass coverslip covered by the Nichoid microscaffold. The microstructured glass coverslip was externally sealed to the Petri dish bottom through a viscous biocompatible UV-light-curable adhesive, Loctite 3345 (Henkel, Cleveland, OH, USA). The glue was then polymerized by a diode UV lamp (λ = 365 nm, Hamamatsu, Hamamatsu city, Japan) by performing three irradiation cycles of 30 s each.

### 2.2. Animal Procedures

P1-4 C57BL6 mice were used in this study to obtain brain primary cells that were then cultured. Procedures involving animals and their care were in accordance with national and international laws and policies (EU Directive 2010/63/EU for animal experiments). The Mario Negri Institute for Pharmacological Research—IRCCS (Milan, Italy) Animal Care and Use Committee (IACUC) approved the study, which was conducted according to the institutional guidelines and in compliance with Italian law. The scientific project was approved by the Italian Ministry of Health (project number 9F5F5.N.WWZ).

### 2.3. Primary Astrocyte Culture

Primary astrocytes were obtained from P3-P4 C57BL6 pups. Hippocampi were dissected in Hanks’ balanced salt solution (HBSS), incubated with 200 U of trypsin (15 min, 37 °C), and mechanically dissociated in culture media (Dulbecco’s modified Eagle’s medium, 100 U/mL penicillin, 100 μg/mL streptomycin, and 10% fetal bovine serum). Cells were centrifuged (7 min, 1500 rpm, RT), and the pellet was resuspended in culture medium and seeded (300,000 cells/mL) onto T25 flasks pre-coated with 25 μg/mL poly-d-lysine. After 7 days, in order to produce astrocyte cultures free of microglia, oligodendrocytes, and neurons, the flasks were shaken at 250 rpm for 12 h (37 °C). After that, cells were split and seeded onto IBIDI chamber slides (150,000 cells/mL) or seeded on the Nichoid substrate (1,000,000 cells/mL) pre-coated with 25 g/mL poly-D-lysine.

### 2.4. Primary Neuronal Culture

Primary neuronal cultures were obtained from P1-P2 C57bl6 pups as described in Sclip et al., 2013 [32], with minor modification. In brief, after dissection, hippocampi were incubated with 200 U of papain (P3125, Sigma Aldrich, St. Louis, MO, USA) (30 min, 34 °C), with trypsin inhibitor (T-9253, Sigma Aldrich, St. Louis, MO, USA) (45 min, RT) and subsequently mechanically dissociated. Neurons were seeded on the astrocyte layer both on ibidi flat chamber slides (300,000 cells/mL) and on the Nichoid substrate (1,500,000 cells/mL). The plating medium was B27/neurobasal-A (Gibco-Invitrogen) supplemented with 0.5 mM glutamine (Gibco-Invitrogen), 100 U/mL penicillin, and 100 μg/mL streptomycin (Gibco-Invitrogen).

### 2.5. Set-Up of the Co-Culture

Nichoids were cleaned with 70% ethanol for 30 min, washed three times with sterile water, and then sterilized with UV over-night. Both the Nichoid substrates and ibidi chamber slides were coated with poly-D-Lysine.

**Figure 1:**
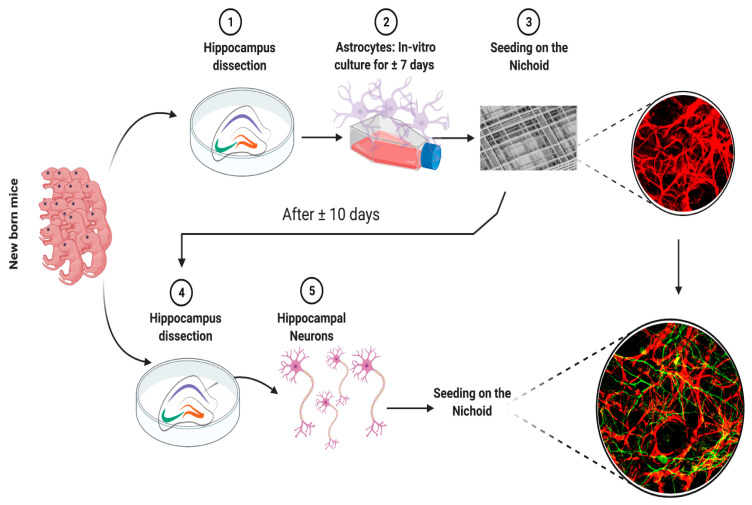
Schematic illustration of the primary hippocampal astrocyte—neuron co-culture set-up in the 3D synthetic microscaffold Nichoid.

The co-culture was formed to stratify the two different cell types. The first cell type plated on both the 2D and 3D substrates was hippocampal astrocytes. After approximately 10 days, astrocytes formed a monolayer in the 2D ibidi chamber and colonized the 3D volume of the Nichoid structure. At this point, hippocampal neurons were plated on the astrocytes. The analyses were performed at 21 days from the neuron plating date (21DIV), at which time cells were well-differentiated and mature (Figure 1).

After their use, Nichoids were decellularized by incubating them with 200 U of trypsin at 37 °C overnight, washed with sterile water and 70% ethanol, and then sterilized again with UV.

### 2.6. Immunofluorescence

Immunofluorescence experiments were performed according to Colnaghi et al., 2019 [33]. Briefly, 2D and 3D co-cultures were fixed in 4% paraformaldehyde (PFA) and a 2% sucrose solution for 30 min, followed by permeabilization with pH 7.4 phosphate-buffered saline (PBS) containing 0.5% Triton X-100 for 1 min. Co-cultures were first blocked for 1 h in PBS containing 1% BSA and then were incubated overnight at 4 °C with primary antibodies in PBS containing 1% BSA and 0.2% Triton X-100. The following antibodies were used: microtubule-associated protein 2 (Map2) (Abcam, AB5392, Cambridge, UK), GFAP (Dako, Z0334), drebrin (Vinci-Biochem, BSR-M05530, Vinci, Italy), PSD95 (Cayman, 10011435), and synaptophysin (Sigma, 041M4782). Cells were finally incubated with secondary antibodies (AlexFluor Antibody, Thermo Fisher Scientific, Waltham, MA, USA) for 1 h at room-temperature. Then, 2 mg/mL Hoechst (Thermo Fisher Scientific, Waltham, MA, USA, 33342) was used to stain nuclei. ProLong Glass Antifade Mountant (Thermo Fisher Scientific) was used as a mounting agent.

### 2.7. Confocal Microscopy

The confocal microscopy technique was adapted from a protocol previously described [33]. Briefly, samples were acquired using a Nikon A1 confocal microscope, and images were collected using 20× and 40× objectives with stack thicknesses of between 0.24 and 0.48 μm. The obtained images were processed with the Nikon NIS-Elements platform and the open-source platform for biological image analysis [34].

### 2.8. Scanning Electron Microscopy (SEM)

For SEM observations, cells cultured on ibidi as well as on Nichoid were fixed in 0.5% glutaraldehyde for 1 h at 4 °C, washed in cacodylate buffer, and post-fixed with 1% OsO4 for an additional hour. The fixed specimens were dehydrated through a series of passages in increasing ethanol baths and dried in pure hexamethyldisilazane (HMDS, Fluka Chemie AG, Buchs, Switzerland). The samples were mounted on stubs and coated with gold in a sputter coater (Agar Scientific Ltd., Stansted, England). The coated specimens were observed by SEM using secondary electron detection (Supra55, Carl Zeiss GmbH, Jena, Germany).

### 2.9. Neuronal Protrusion Analysis

The 2D and 3D co-cultures were analyzed at DIV21 for the manual counting of protrusions, adapting a protocol from Drisaldi et al., 2015 [35]. Briefly, 40 μm long dendritic segments were selected and imaged using a Nikon A1 confocal microscope. Two operators performed a double-blind protrusion count. For each group, we analyzed 20 dendritic segments.

### 2.10. Statistical Analysis

D’Agostino/Pearson, Shapiro–Wilk and KS normality tests were performed. Filopodia counts were subjected to a non-parametric Mann–Whitney *t*-test. The data were expressed as means ± SEM with statistical significance at *p* < 0.05. The statistical analysis was performed with the Graph Pad Prism 6 program.

## 3. Results

### 3.1. Co-Cultures of Astrocytes and Neurons in 2D vs. 3D Microscaffold

The microscaffold Nichoid was initially designed to create a microarchitecture mimicking the structural arrangement of a native 3D stem cell niche [28,36,37]. As a second step in the investigation of its potential uses in biotechnology, here we investigated how the 3D Nichoid would allow the creation of brain tissue using post-mitotic neurons and glial cells (mitotic cells). We therefore co-cultured primary hippocampal astrocytes and neurons from neonatal mouse brains to investigate whether the 3D scaffold allows the post-mitotic neurons to migrate within the scaffold and develop a neuronal morphology and their intercellular connections. For this, primary brain cells were isolated directly from neonatal mouse nervous tissue. Different from cell lines (including stem cells), these isolated brain cells maintain the features of their tissue of origin without requiring lengthy differentiation protocols. For this, they are a relevant and widely used tool for the study of physiological and pathological aspects in the field of neuroscience. The culture of neuronal cells is, however, challenging since differentiated neurons are post-mitotic and therefore do not undergo cell division like cell lines or stem cells.

To determine whether the Nichoid could be a feasible scaffold for 3D primary brain cell cultures, we first seeded astrocytes obtained from P2-4 C57BL6 pups to define the minimum cell density necessary to guarantee a sufficient number of contacts between the cells as well as the astrocytes’ possibility of cell division. After 10 days of in vitro culture, we seeded the primary neurons on top of the layer of astrocytes.

Since astrocytes and neurons in the rodent central nervous system (CNS) appear to be regional and cortical-layer-specific in terms of the numbers and dimensions of axons and dendrites as well as functions, we decided to use mouse astrocytes and neurons from the same brain area: the hippocampus (Figure 1).

**Figure 2:**
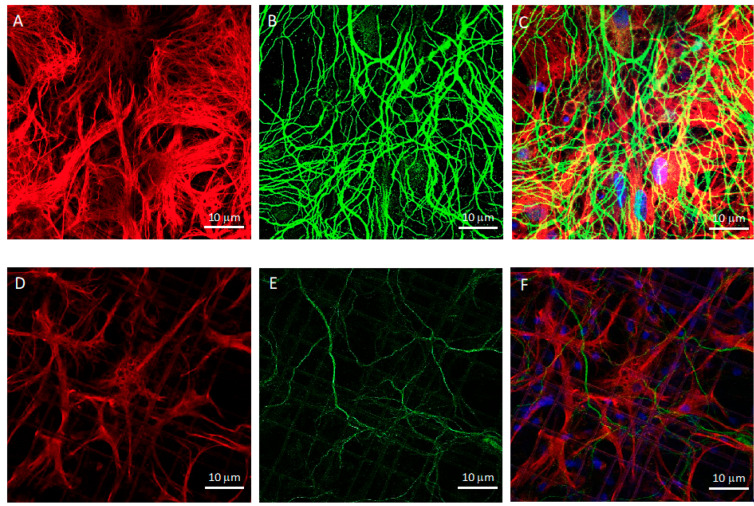
Confocal microscopy of DIV21 hippocampal astrocyte and neuron co-cultures in 2D flat culture and in the 3D synthetic microscaffold Nichoid. (**A**–**C**) 2D co-cultures formed by (**A**) hippocampal astrocytes, marked with GFAP in red, and (**B**) hippocampal neurons, marked with MAP2 in green. Nuclei were stained with Hoechst, in blue. (**D**–**F**) Co-culture in the 3D Nichoid formed by (**D**) hippocampal astrocytes, marked with GFAP in red, and (**E**) hippocampal neurons, marked with MAP2 in green. Nuclei were stained with Hoechst, in blue. Images were taken with a 100× (oil immersion) objective. Scale bar of 10 μm.

At DIV21, upon fixation, we stained both co-cultures with specific antibodies raised against a set of basic cell markers to compare the cell morphology and colonization of the 3D and 2D environments with confocal microscopy. Figure 2 presents z-projections of immunofluorescence acquisitions showing astrocytes marked with GFAP (red), neurons marked with MAP2 (green), and nuclei in blue in 3D Nichoids (Figure 2D–F) and on the 2D flat substrate (Figure 2A–C). From qualitative observations of the confocal microscopy images, both cell types appeared well-differentiated after 21 days in vitro. In the 2D co-cultures, astrocytes formed a confluent monolayer, with cells covering the available surface. In addition, the astrocyte morphology seemed different between the 2D and 3D cultures (Figure 2); in fact, in the Nichoid, the ramifications appeared more defined, and their shapes were more radial (Figure 2A–D).

**Figure 3:**
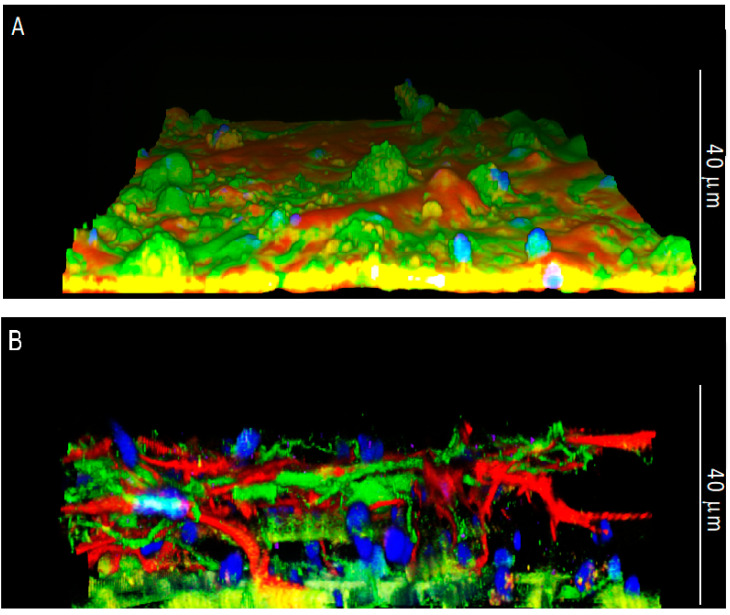
Confocal microscopy of DIV21 hippocampal astrocyte and neuron co-cultures in 2D and in the 3D synthetic microscaffold Nichoid. Lateral views of (**A**) 2D and (**B**) 3D co-cultures in which hippocampal astrocytes were marked with GFAP in red and neurons were marked with MAP2 in green. Nuclei were stained with Hoechst in blue. Images were taken with a 100× (oil immersion) objective.

To determine the 3D occupancy of the cultures, we next took confocal images using a 100× objective, and we imaged the samples with planes in increments of 0.25 μm to be able to recreate a 3D image. Figure 3 represents such reconstruction, where it is clearly visible that cells in the Nichoid occupied all the 3D space.

**Figure 4:**
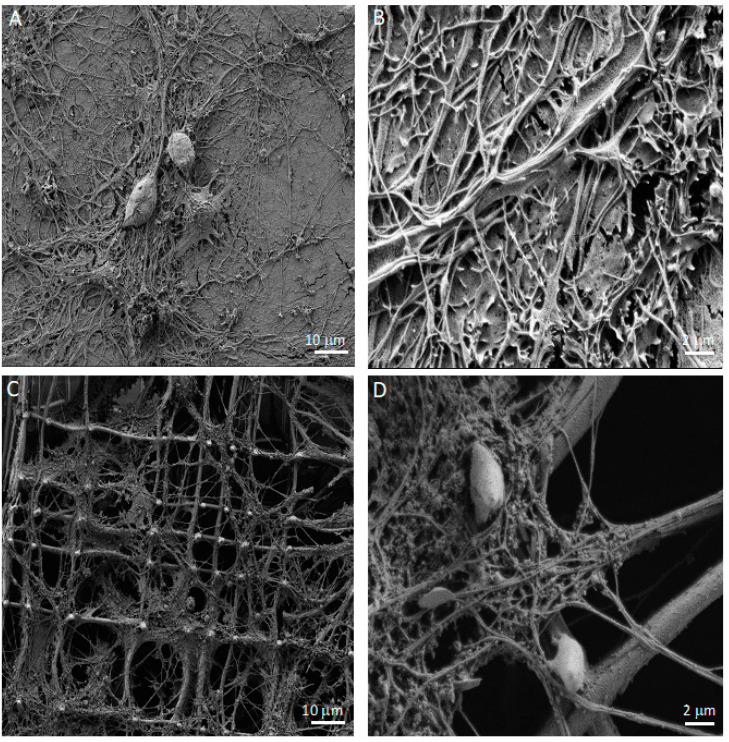
SEM microscopy of DIV21 hippocampal astrocyte and neuron co-cultures in 2D and in the 3D synthetic microscaffold Nichoid. (**A**) The 2D hippocampal astrocyte and neuron co-culture. Scale bar of 10 μm. (**B**) Higher magnification of 2D hippocampal astrocyte and neuron co-culture. Scale bar of 2 μm. (**C**) The 3D hippocampal astrocyte and neuron co-culture in the Nichoid. Scale bar of 10 μm. (**D**) Higher magnification of 3D hippocampal astrocyte and neuron co-culture in the Nichoid. Scale bar of 2 μm.

To further characterize the cell morphology in the Nichoid compared to the 2D culture, we examined the co-cultures with a scanning electron microscope (SEM). On the 2D support, astrocytes displayed well-extended microvillar structures (Figure 4A,B) with small lamellipodia on their cell surfaces, indicating the fluidity and motility of cells, similar to what Führmann and colleagues showed [38]. Under this condition, the neurons were forced to grow on top of the astrocytic monolayer, creating a uniform and dense network of neurites from which only the somas protruded. The neuronal somas had a granular appearance, while the neurites were mostly smooth (Figure 4A,B). In contrast, in the 3D co-culture, a fraction of cells sedimented on the bottom of the Nichoid, but a great number of cells were attached to the mesh of the 3D structure and colonized the entire thickness of the microscaffold. Astrocytes formed a sort of sail. Growing between the meshes of the scaffold (Figure 4C), they displayed more lamellipodia and displayed a more rippled surface. Compared to the 2D co-culture, neurons in the 3D microscaffold could self-organize into a three-dimensional configuration, growing and contacting the meshes of the microscaffold, other neurons, and/or astrocytes (Figure 4C,D). Although neurons preferentially followed the distribution of astrocytes, they were also found to extend isolated neurites crossing the walls and lattice of the Nichoid. Contrary to the 2D culture, in the 3D culture neuronal somas presented a smooth surface while their neurites displayed more protrusions (Figure 4C,D).

### 3.2. Neurons: Spines/Protrusions

An important aspect of this 3D co-culture model is the long-lasting viability that allows the full differentiation and maturation of astrocytes and neurons in the Nichoid after 21 days of culture in vitro. Neurons are post-mitotic cells that are unable to divide and characterized by the ability to make connections. The complete maturation of these cells is represented by the presence of neuronal protrusions, including filopodia and dendritic spines.

**Figure 5:**
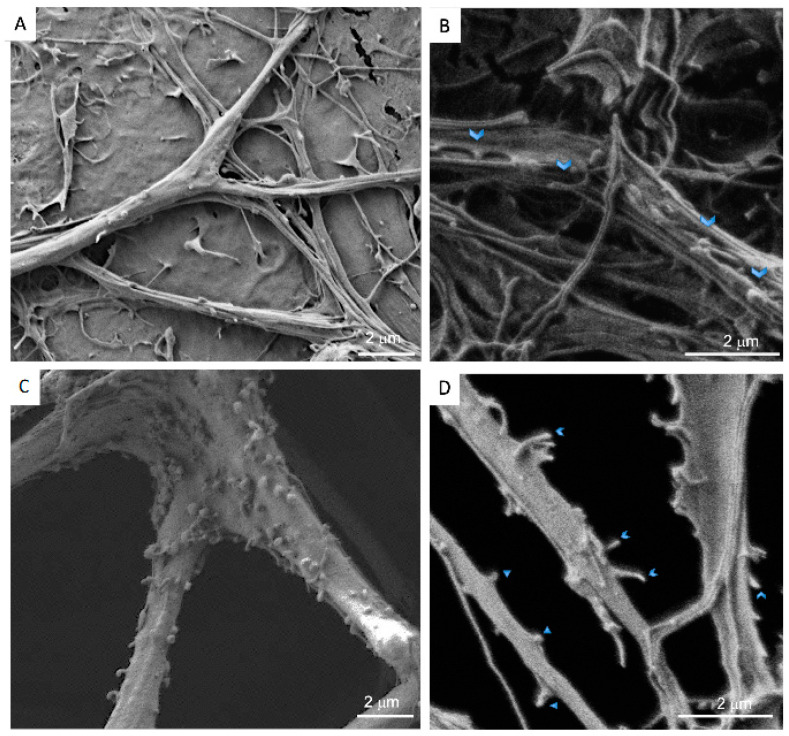
SEM microscopy of DIV21 hippocampal neurons co-cultured with astrocytes in 2D and in the 3D synthetic microscaffold Nichoid. (**A**) The 2D hippocampal astrocyte and neuron co-culture. Scale bar of 2 μm. (**B**) Higher magnification of 2D hippocampal astrocyte and neuron co-culture. Filopodia and spines were indicated by arrows. (**C**) The 3D hippocampal astrocyte and neuron co-culture. Scale bar of 2 μm. (**D**) Filopodia (arrow) and spine (triangle) numbers increased in 3D hippocampal astrocyte and neuron co-cultures. Scale bar of 2 μm.

Using SEM, we compared the morphology of the protrusions between the 2D and 3D neurons (Figure 5A,B). After 21 days, the neurons in both the 2D and 3D cultures showed mature protrusions belonging to two categories: filopodia and mature spines (Figure 5C,D).

### 3.3. Spine/Protrusion Counts in 2D vs. 3D

**Figure 6:**
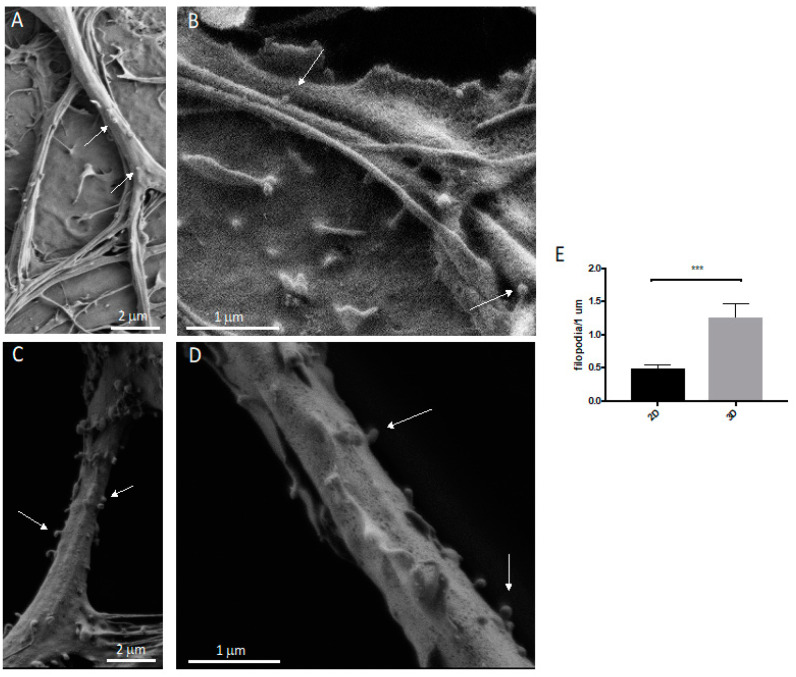
SEM microscopy and protrusion quantification of DIV21 hippocampal neurons co-cultured with astrocytes in 2D and in the 3D synthetic microscaffold Nichoid. (**A**) The 2D hippocampal astrocyte and neuron co-culture. Scale bar of 2 μm. (**B**) Higher magnification of 2D hippocampal astrocyte and neuron co-culture. Scale bar of 1 μm. Arrows indicate the protrusions. (**C**) The 3D hippocampal astrocyte and neuron co-culture in the Nichoid. Scale bar of 2 μm. (**D**) Higher magnification of 3D hippocampal astrocyte and neuron co-culture in the Nichoid. Scale bar of 1 μm. Arrows indicate examples of filopodia that were considered in the quantification. (**E**) Results of filopodia counts. Conditions were compared using *t*-tests. Statistical significance: *** *p* < 0.001. Data are the means ± SEM of 20 dendrites from three independent conditions.

Next, we investigated whether the use of the Nichoid microscaffold influences the dendritic spines’ density by quantifying these protrusions. Using a higher magnification of SEM images, as shown in Figure 6E, neurons in the 3D co-culture displayed a larger number of protrusions (both filopodia and dendritic spines) compared to neurons in the 2D co-culture (*p* < 0.001; 2D: mean 0.4899, SD 0.2159; 3D: mean 1.265, SD 0.8028; Figure 5A,B). The spine density (number of spines per neurite length) changes in physiological mechanisms as well as during several pathological conditions, playing a crucial role in synaptic plasticity, learning, and memory; a reduced spine number can reflect changes in the strength of synaptic transmission, and it is often associated with cognitive impairment and neurodegeneration.

The 3D Nichoid microscaffold strongly modified mechanotransduction processes, influencing not only the cytoskeletal organization but also the nuclear morphology and permeability, which may change cell fate and behavior in the absence of exogenous chemical signals [39,40]. Similarly, the 3D Nichoid induced an increase in the protrusion number, equal to 38%, in the 3D compared to the 2D co-culture (Figure 6).

**Figure 7:**
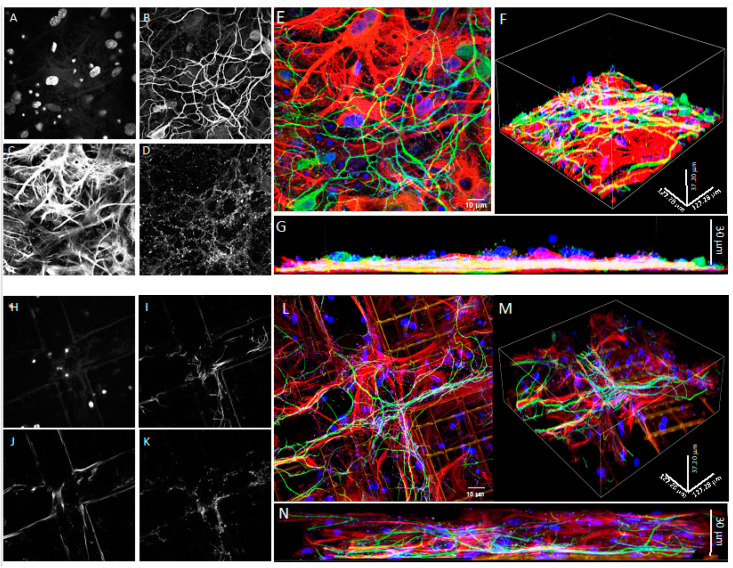
Confocal microscopy of DIV21 hippocampal co-cultures of astrocytes and neurons in 2D and in the 3D synthetic microscaffold Nichoid. (**A**–**D**) Grayscale images from horizontal sections of 2D hippocampal co-cultures of astrocytes and neurons marked with Hoechst (**A**), MAP2 (**B**), GFAP (**C**), and drebrin (**D**). (**E**) Merged fluorescence image of 2D hippocampal co-cultures with Hoechst in blue, MAP2 in green, GFAP in red, and drebrin in violet. (**F**,**G**) Lateral views of 2D hippocampal astrocyte and neuron co-cultures displayed in (**E**). (**H**–**K**) Grayscale horizontal sections of 3D hippocampal astrocyte and neuron co-cultures marked with Hoechst (**H**), MAP2 (**I**), GFAP (**J**), and drebrin (**K**). (**L**) Merged fluorescence image of 3D hippocampal astrocyte and neuron co-cultures with Hoechst in blue, MAP2 in green, GFAP in red, and drebrin in violet. (**M**,**N**) Lateral sections of 3D hippocampal astrocyte and neuron co-cultures displayed in L. Images were taken with a 100× (oil immersion) objective.

**Figure 8:**
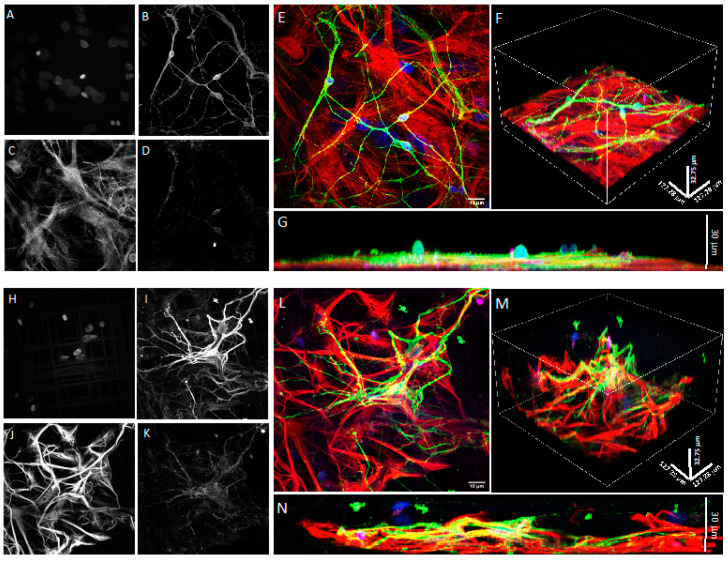
Confocal microscopy of DIV21 hippocampal co-cultures of astrocytes and neurons in 2D and in the 3D synthetic microscaffold Nichoid. (**A**–**D**) Grayscale images from horizontal sections of 2D hippocampal astrocyte and neuron co-cultures marked with Hoechst (**A**), MAP2 (**B**), GFAP (**C**), and PSD95 (**D**). (**E**) Merged fluorescence image of 2D co-culture with Hoechst in blue, MAP2 in green, GFAP in red, and PSD95 in violet. (**F**,**G**) Lateral views of the 2D co-cultures displayed in (**E**). (**H**–**K**) Grayscale images of horizontal sections of 3D hippocampal co-cultures of astrocytes and neurons marked with Hoechst (**H**), MAP2 (**I**), GFAP (**J**), and PSD95 (**K**). (**L**) Merged fluorescence image of the 3D hippocampal co-cultures with Hoechst in blue, MAP2 in green, GFAP in red, and PSD95 in violet. (**M**,**N**) Lateral view of 3D co-cultures displayed in L. Images were taken with a 100× (oil immersion) objective.

**Figure 9:**
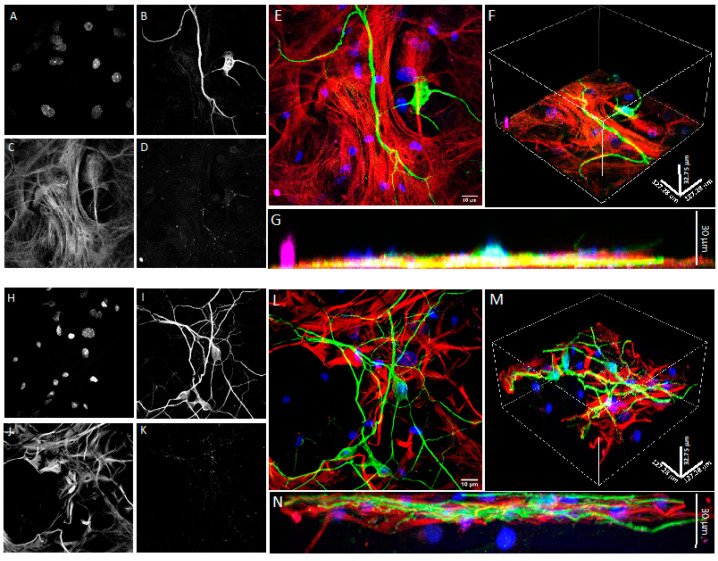
Confocal microscopy of DIV21 hippocampal astrocyte and neuron co-cultures in 2D and in the 3D synthetic microscaffold Nichoid. (**A**–**D**) Grayscale images of horizontal sections of 2D hippocampal co-cultures of astrocytes and neurons marked with Hoechst (**A**), MAP2 (**B**), GFAP (**C**), and synaptophysin (**D**). (**E**) Merged fluorescence image of 2D hippocampal co-cultures with Hoechst in blue, MAP2 in green, GFAP in red, and synaptophysin in violet. (**F**,**G**) Lateral sections of 2D hippocampal astrocyte and neuron co-cultures displayed in (**E**). (**H**–**K**) Grayscale horizontal sections of 3D hippocampal astrocyte and neuron co-cultures marked with Hoechst (**H**), MAP2 (**I**), GFAP (**J**), and synaptophysin (**K**). (**L**) Merged fluorescence image of 3D hippocampal astrocyte and neuron co-cultures with Hoechst in blue, MAP2 in green, GFAP in red, and synaptophysin in violet. (**M**,**N**) Lateral sections of 3D hippocampal astrocyte and neuron co-cultures displayed in L. Images were taken with a 100× (oil immersion) objective.

To further assess whether the use of the Nichoid microscaffold alters spine differentiation and maturation, we used immunological markers [33]. Upon fixation, the 2D and 3D co-cultures were stained with GFAP, MAP2, DAPI, and drebrin (an F-actin-binding protein highly enriched on the postsynaptic terminals of excitatory synapses also playing a key role in spine shaping) [41] or PSD95, the most abundant scaffold of the active zone, anchoring the NMDA and AMPA receptor subunits in the postsynaptic element. The 3D reconstruction of confocal images shows that neurons in the Nichoid co-culture (Figure 7E,F) spread throughout the entire 30 μm thickness of the microscaffold compared to the flat culture (Figure 7B,C). In both conditions neurons were abundantly marked with drebrin (Figure 7E,F), PSD95 (Figure 8A–F) and synaptophysin (Figure 9A–F), demonstrating that both the 2D and 3D co-cultures displayed mature dendritic spines.

## 4. Discussion

For the first time, we applied the Nichoid technology to culture primary brain cells, with the aim of proposing a new method for 3D primary brain cultures superior to the classical 2D co-culture method but with similar costs, time, effort, and feasibility. Along these lines, we find that compared to 2D cultures our 3D ones display more protrusion/spines and subcellular compartments regulated by cytoskeletal changes that strongly influence both connectivity and excitability. Furthermore, the immunological characterization of their maturation showed abundant labeling for both drebrin, PSD95, and synaptophysin staining in 2D as well as 3D.

Upon seeding in the 3D microscaffold, we expected that the majority of cells would fall by sedimentation, primarily distributed at the bottom of the Nichoid. However, a large number of astrocytes anchored to the walls of the Nichoid, also colonizing the empty spaces between the structural micro trusses that form the microscaffold architecture. Instead, in line with the 2D culture, neurons followed the distribution of the astrocytes by growing on top of them. These differences could be due to the structure of the Nichoid that strongly modifies the cell adhesion configuration and reflected cytoskeletal organization, as we have previously demonstrated for mesenchymal stem cells and neural progenitors [42,43]. In addition, it seems that astrocytes in 3D culture self-organize in well-differentiated domains, as happens in the brain parenchyma, as opposed to the 2D substrate where they were mainly overlapping each other (Figure 2A–D). In the 2D culture, astrocytes formed a confluent monolayer, with cells covering the available surface (Figure 3A). On the contrary, cells seeded on the 3D Nichoid penetrated the internal structure of the microscaffold (Figure 3B). Concerning neurons, their morphology appeared similar between the 2D and 3D cultures. However, in the 3D culture, neurons were also contacting not only the surrounding neurons and astrocytes but also the niche’s 3D structure (Figure 3B).

In the 2D co-cultures, astrocytes displayed well-extended microvillar structures (Figure 4B) with small lamellipodia on their cell surfaces, indicating the fluidity and motility of the cells [8]. On the other hand, neurons were forced to grow on top of the astrocytic monolayer, creating a uniform and dense network of neurites in which only the somas protruded from the flat carpet. The neuronal somas had a granular appearance, while the neurites were mostly smooth (Figure 4A,B). In contrast, in the 3D co-culture, a fraction of the cells sedimented on the floor of the Nichoid, but a great number of cells were attached to the mesh of the 3D structure and colonized the entire thickness of the microscaffold. Astrocytes formed a sort of sail. Growing between the meshes (Figure 4C), they displayed more lamellipodia structures and a more rippled surface. Compared to the 2D co-culture, neurons in the 3D microscaffold could self-organize into a three-dimensional configuration, growing and contacting the meshes of the microscaffold as well as other neurons and/or astrocytes (Figure 4C,D). Although neurons preferentially followed the distribution of astrocytes, they were also found to extend isolated neurites crossing the walls and lattice of the Nichoid. Contrary to the 2D culture, in the 3D culture the neuronal somas presented a smooth surface while their neurites displayed more protrusions (Figure 4C,D).

The morphogenesis of the dendritic spine (as well as its dysmorphogenesis) is regulated by cytoskeletal re/organization within the neuron but is also influenced by the astrocytes; in fact, structural changes in astrocytes regulate the degree of neuron–glial communication at the synapse level [44]. Since changes in astrocytic processes are typically accompanied by changes in the morphology of the spines, it is also possible that the opportunity to grow in a 3D environment, which is more flexible and less static compared to a flat 2D environment, influences the interactions and relationships between astrocytes and neurons, stimulating a higher number of filopodia protrusions.

What differentiates our model from those already available is the relative simplicity of production and the use of the device since no special equipment is required for the read-out, utilization, and reproducibility. In fact, as opposed to the hydrogels that mimic the natural ECM but are limited in scale because of long-term storage issues, stability, and batch-to-batch variability [45], the Nichoid is composed of inert material, not degraded by cells and, thanks to the manufacturing methods, always reproducible compared to the initial design. All this allows the growth and proper development of primary cells that, different from iPSCs and organoids that require months of culture before being ready for experiments, only need a few weeks to mature. Moreover, the 3D cultures are comparable to the classic 2D ones in terms of the costs and time of preparation.

Overall, the development of this in vitro model based on the inert Nichoid microscaffold mimicking the 3D environment of the brain parenchyma offers the possibility to study adult hippocampal neurons and astrocytes and their cell–cell relationship in physiological and pathological conditions. The astrocyte and neuron co-culture 3D model could be useful to study the molecular and cellular mechanisms governing synaptic function in physiological conditions and will offer the possibility to mimic pathological conditions, inducing, with different stress stimuli, synaptic dysfunction, the first neurodegenerative event, offering the possibility to investigate this mechanism underlying the initial step of many different brain diseases. This 3D Nichoid co-culture is the first step to generating a model with the final aim to identify the pathological mechanisms/pathways/key modulators underlying synaptopathy. It can also be used to study neuronal death and deeply investigate mechanisms/events in different brain diseases.

Importantly, we underline the lack of adequate in vivo preclinical models of synaptic dysfunction and the need for a new simple and low-cost in vitro model to analyze the mechanisms/pathways that govern the spine that is available for drug screening against synaptic failure. Suggestively, once a key player in plasticity/dysfunction in the 3D model is identified, it will be studied with different pharmacological treatments and drug screening in vivo. This is because the 3D culture model offers an environment that better represents the brain architecture compared to traditional 2D ones. It also offers a higher surface area that can better accommodate cell differentiation, maturation, growth, and migration.

Future developments will focus on exploiting these results to implement an even more complete 3D co-culture, adding adult microglia to investigate all the main cell types involved in synapse function/dysfunction. In this context, this in vitro model will also be suitable for large-scale drug screening against synaptic dysfunction and in general against brain diseases. Another important future direction will be to develop systems that will allow measuring the electrical potentials in the 3D Nichoid. This will permit researchers to define the networking functionality. Two different methodologies are currently under revision for this issue.

## Data Availability

Data are available upon reasonable request to the corresponding author.
